# Accelerometry: A Feasible Method to Quantify Physical Activity in Ambulatory and Nonambulatory Adolescents with Cerebral Palsy

**DOI:** 10.1155/2012/329284

**Published:** 2012-06-26

**Authors:** Jan Willem Gorter, Stephen G. Noorduyn, Joyce Obeid, Brian W. Timmons

**Affiliations:** ^1^CanChild Centre for Childhood Disability Research, McMaster University, 1400 Main Street West, IAHS Building, Room 408, Hamilton, ON, Canada L8S 1C7; ^2^Health Research Methodology Program, McMaster University, Hamilton, ON, Canada L8S 4K1; ^3^Child Health & Exercise Medicine Program, Department of Pediatrics, McMaster University, 1280 Main Street West, HSC 3N27G, Hamilton, ON, Canada L8S 4K1

## Abstract

*Objective*. To determine the feasibility of physical activity monitoring in adolescents with cerebral palsy (CP). *Methods*. A convenience sample of ambulatory and non-ambulatory adolescents (*N* = 23; 17 males, 6 females; mean age 13.5 y, SD 2.6 y; Gross Motor Function Classification System (GMFCS) distribution: *n* = 9 Level I, *n* = 5 Level II, *n* = 5 Level III, *n* = 4 Level IV) was recruited. Physical activity (PA) was objectively assessed using the *ActiGraph* GT1M activity monitor. Discomfort or adverse effects of wearing the accelerometers were recorded by participants. Levels of physical activity were determined as total PA, light PA (LPA), moderate PA (MPA), moderate-to-vigorous (MVPA), and vigorous PA (VPA) using cut-points recently validated for CP. *Results*. Most participants showed little reluctance. Mean daily MVPA for all participants was 30.7 minutes (SD 30.3), which corresponded to 2.7 (SD 2.4) minutes of MVPA per hour or 4.5% (SD 3.9) of the total monitoring time. Total PA and MVPA were greatest in ambulatory youth (GMFCS levels I and II) compared with youth who use a walking aid or wheelchair (GMFCS levels III and IV) (*P* < 0.05). *Conclusion(s)*. The results support the use of the accelerometer as a feasible and useful measure of activity in ambulatory and nonambulatory adolescents with CP.

## 1. Introduction 

Impaired motor function is the hallmark of cerebral palsy (CP). As a result, children and adolescents with CP are at particular risk for inactivity and the associated negative health impacts (e.g., obesity with exacerbated cardiovascular dysfunction) [1–3]. Not surprisingly, physical activity for children and adolescents with CP is commonly prescribed in clinical practice [4]. However, recent intervention studies have not been able to show a sustained increased activity level in the home, school, or community settings [5, 6]. 

We are currently designing the Stay-FIT intervention study to implement and evaluate a community-delivered physical activity programme for youth with CP across the severity spectrum [7]. This intervention study is part of a translational research programme focusing on physical activity in individuals with CP (http://www.canchild.ca/en/ourresearch/stay_fit.asp). The Stay-FIT programme will examine issues of physical activity covering several domains described by the World Health Organization's International Classification of Functioning (ICF) [8], ranging from cardiovascular function and structure [9] to performance in physical activity. This programme reflects the recent paradigm shift in therapy for youth with CP by using physical activity as both the focus of intervention and the primary outcome. 

In order to design these future interventions, an acceptable outcome measure for physical activity is needed. Accelerometer-based activity monitoring provides an excellent measure of daily physical activity as it can be used to measure the amount, intensity, and pattern of both activity and sedentary behavior [10–13]. In 2010, Capio et al. published a validation study on the use of a uniaxial accelerometer (MTI) to monitor activity in 31 ambulatory children with CP, Gross Motor Function Classification System (GMFCS) levels I-III [11]. Although this study did not examine the feasibility of an accelerometer systematically, the authors reported that the participants did not manifest any indications of intolerance with the device. The accelerometer was able to capture raw activity volume in unstructured free play and in six structured activities of increasing intensities including sitting, walking, and jogging. The validity of this device as a measure of PA volume was confirmed by its linear association with measured heart rate and observed PA. GMFCS levels, however, explained 0 and less than 1% of the variance in activity during structured and free play activities, respectively. It must be noted that the participants' accelerometer data demonstrated a large degree of variance, as shown by the high standard deviations. Therefore, the authors deemed it inappropriate to use regression equations to predict an activity cut-point for MVPA. In 2011, a study using the *ActiGraph* 7164 accelerometer was published by Clanchy et al. including mainly ambulatory children and adolescents (mean age 12.6 ± 2.0 years) classified at GMFCS level I or II withonly a small number of GMFCS III subjects [12]. This study showed that the *ActiGraph* 7164 is able to differentiate between different intensities of walking in children and adolescents with CP. The validity of the *ActiGraph* accelerometer as a measure of PA was confirmed by using directly measured oxygen uptake as a criterion measure. Unfortunately the data did not provide sufficientpower to perform meaningful subgroup analyses by GMFCS level. Van den Berg-Emons et al. reported in 2011 on the physical activity measurement in adults with various diagnoses [10]. In this study a small number ofnonambulatory adults with CPwere included (*n* = 4) withlimited wear time of 2 consecutive days (48 hours). The authors were able to show that activity volume and intensity could be measured in nonambulatory adults with CP during various activities including wheelchair driving. To our knowledge, there have been no studies to date that have evaluated the psychometric properties of accelerometer-based assessments of habitual physical activity in non-ambulant adolescents with CP (GMFCS level IV and V) [13]. 

Despite the growing interest in physical activity and the use of accelerometers in the CP population, there remains a gap in our knowledge on assessment of habitual physical activity at home, at school, and in the community in adolescents with CP, particularly for those with more severe functional limitations [11–13]. In this paper we present the results of the Stay-FIT *pilot* study that was developed to test the feasibility of accelerometry for use in ambulatory and nonambulatory adolescents with CP. 

## 2. Materials and Methods

### 2.1. Participants

Adolescents were recruited through regional spasticity and teenager-transition clinics of a university medical center. Participants met the following inclusion criteria: (1) age between 10 and 20 years; (2) a definite diagnosis of CP; (3) a GMFCS level I, II, III, or IV. Between October 2009 and January 2011, 31 children and adolescents with CP were identified and agreed to be contacted with respect to study participation. Of these 31, four candidates opted not to participate, three candidates agreed to participate but later withdrew from the study, and one participant was diagnosed with an acquired brain injury and was subsequently excluded. As a result, 23 children and adolescents (17 males, 6 females; mean age 13.5 y, SD 2.6 y) completed the study, of which nine were classified at GMFCS level I, five level II, five level III, and four level IV. For classification of severity we used the GMFCS Expanded and Revised version (GMFCS-ER) that has excellent interrater reliability for use in adolescents (12–18 years) [14]. All parents/guardians and participants provided written consent/assent to participate in this study approved by the Faculty of Health Sciences/Hamilton Health Sciences Research Ethics Board, Hamilton, Canada.

### 2.2. Assessment of Physical Activity

Habitual physical activity was objectively assessed using the *ActiGraph* GT1M activity monitor. This device was chosen for the purposes of this study for its ability to measure activity over a relatively prolonged period while remaining unobtrusive. The *ActiGraph* GT1M accelerometer weighs 27 g with dimensions of 3.8 × 3.7 × 1.8 cm (i.e., about the size of a matchbox) and measures and records acceleration in the vertical plane ranging from ~0.05 to 2 G in magnitude. The acceleration is sampled and digitized by a 12-bit analog-to-digital converter at a rate of 30 Hz. This signal is passed through a digital filter that eliminates nonhuman motion and then stored in user-defined intervals (i.e., epochs). Given the brief, intermittent, and spontaneous nature of activity reported in youth, physical activity was recorded in 3-second recording intervals or epochs [15].

### 2.3. Procedure

**Figure 1:**
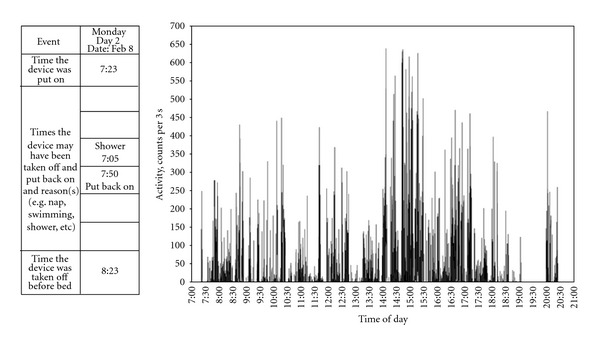
An example of a typical monitoring day for one participant with CP (female, 11 years of age, GMFCS level I).The table is a sample from the participant*ʼ*s log book in which they were asked to record the times the device was put on and taken off for each of the monitoring days. The figure to the right represents the accelerometer output for the corresponding day. No activity was recorded by the device from 7:05 to approximately 7:56 pm, which corresponds to the time the participants indicated they removed the device.

Participants were instructed to wear the accelerometer over the right hip during all waking hours for seven consecutive days, except when engaging in water activities so as not to damage the equipment. A seven-day period was selected to ensure that measured activity was representative of habitual levels of physical activity [16]. A log book was kept with the intent of tracking all times (and reasons) the device was removed and replaced.  Figure 1 shows an example of a logbook and accelerometer output for a typical monitoring day for one participant. Upon completion of the seven days, log books and accelerometers were obtained from the participants and downloaded for further analysis. The research coordinator discussed any difficulties encountered and concerns that may have arisen with the participant and their parents/guardian.

### 2.4. Physical Activity Analysis

Accelerometer data were visually inspected to ensure that information recorded in the log book corresponded with the accelerometer output. Any activity recorded during periods of nonwear time, as indicated by the participant in the log book, was deleted. Observations of consecutive epochs of “0” counts were considered sedentary time unless otherwise stated in the log book. Only participants with ≥5 hours of monitoring time on ≥4 days were included in the analyses. These criteria were selected to maximize participant inclusion and were based on the minimal allowable time previously used to estimate habitual physical activity [16, 17]. The data were then uploaded to a Microsoft Excel-based Visual Basic data reduction program to determine total monitoring time and total activity. The program also distinguished light physical activity (LPA), moderate physical activity (MPA), moderate-to-vigorous activity (MVPA), and vigorous physical activity (VPA). Activity intensity was examined using the cut-points developed by Clanchy et al. and Evenson et al., which were recently validated for use in children and adolescents with CP [12, 18].

### 2.5. Descriptive Statistics

Levels of physical activity (total, LPA, MPA, VPA, and MVPA) between the four GMFCS levels were compared using one-way analysis of variance (ANOVA) in STATISTICA (StatSoft, Tulsa, Okla., USA). Analyses of covariance (ANCOVA) were also performed with chronological age as the covariate so as to account for the distribution in age among participants. Tukey's honestly significant post hoc tests were performed when necessary. Given the small number of participants in each of the GMFCS levels, Kendall's Tau was used to assess the relationship between MVPA and GMFCS level in SPSS (Version 17.0, Chicago, Ill., USA). Descriptive statistics were used to calculate the proportion of participants meeting the Canadian physical activity guideline recommendations for youth (≥60 min MVPA per day). Statistical significance for all analyses was set at *P* ≤ 0.05.

## 3. Results

### 3.1. Feasibility

We found that refusal to participate was most often based upon the lack of parental enthusiasm and the youth's perception that they might look “different” in various social settings. No participants reported discomfort or adverse effects of wearing the accelerometer throughout the duration of the study. One participant exposed the device to water on day 5 of wear, which resulted in highly erratic data. Therefore, that participant's data from only days 1 to 4 were included in the analyses. 

### 3.2. Physical Activity Levels

**Figure 2:**
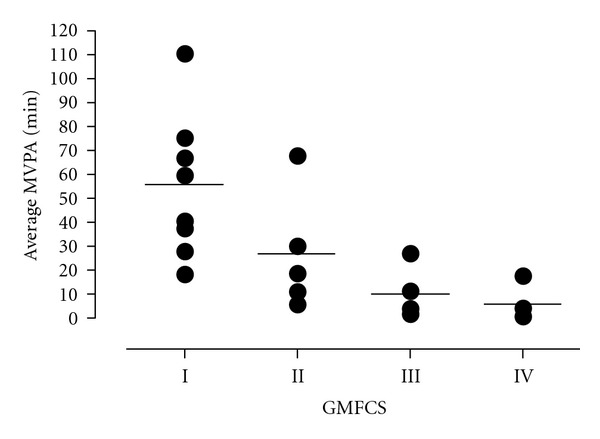
Moderate-to-vigorous activity time in adolescents with CP by GMFCS level. MVPA: moderate-to-vigorous physical activity; CP: cerebral palsy; GMFCS: Gross Motor Function Classification System; min: minutes.

None of the participants were excluded on the basis of failure to wear the accelerometer for the minimum required period (≥5 hours on ≥4 days). On average, the device was worn for 6 of the 7 required monitoring days, with the monitoring period ranging from 540.5 to 859.2 minutes per day (mean ± SD: 707.7 ± 81.2 min). On a daily basis, our participants engaged in (mean ± SD) 89.5 ± 47.1 min of LPA, 17.8 ± 16.9 min of MPA, 12.0 ± 14.4 min of VPA, and 30.7 ± 30.3 min of MVPA. To account for differences in wear time, data were also examined as minutes of activity per hour of monitoring time (Table 1). Both ANOVA and ANCOVA suggested that youth classified at GMFCS level IV presented with lower levels of LPA, MPA, and MVPA compared with level I (*P* < 0.05). Similarly, youth at level III demonstrated lower levels of MPA and MVPA compared with level I. No differences were seen between levels I and II for any intensity, nor were there significant differences between levels II, III, and IV. A significant negative correlation was seen between MVPA, in both minutes/day and minutes/hour, and GMFCS levels (minutes/day: *τ* = −0.65, minutes/hour: *τ* = −0.61, *P* < 0.001, Figure 2). 

## 4. Discussion

This study aimed to assess the ability of accelerometers to measure the duration, intensity, and timing of physical activity in the home, community, and at school of ambulatory and nonambulatory adolescents with CP. The use of the accelerometer worn around the waist to measure habitual physical activity has been shown to be feasible and unobtrusive to participants, including adolescents classified as GMFCS level IV. Our results are in line with recently published data on the *ActiGraph* in children and teenagers up to age 16 years with CP, GMFCS level I and II [11, 12] and add supportive data for its use in adolescents with more functional limitations (GMFCS level III and IV) and older adolescents (up to age 20 years). 

While our findings are promising, a number of limitations both inherent to the accelerometer and related to our analysis should be noted. First, our small sample size may explain the lack of significant findings when comparing activity by GMFCS level. Moreover, the activity cut-points selected were based on the work by Clanchy et al. [12], which was performed only in ambulatory youth with CP. It is unknown whether these same cut-points are applicable in nonambulatory adolescents with CP. Our assessment of activity is further complicated by the use of waist-worn accelerometer in nonambulatory adolescents. More specifically, it is impossible to determine whether the use of these waist-worn accelerometers accurately captured all activity performed by participants classified as GMFCS levels III and IV, particularly in those youth who are able to self-propel their wheelchair. Future work should employ a similar design to that utilized by Clanchy et al. [12] in which the oxygen cost of movement is assessed in conjunction with accelerometer recordings. Finally, it is important to note that the sensitivity of the accelerometer to water presents a particular challenge since aquatic exercise seems to be a preferred activity of youth with CP [19]. Despite this limitation, we believe the ability of accelerometers to measure the duration, intensity, and timing of physical activity in the home, community, and at school outweighs the limitation of missing relatively small amounts of time in a pool. 

With respect to activity monitoring, of the 23 participants, none engaged in ≥60 min MVPA per day recommended in the Canadian Physical Activity Guidelines for youth on a daily basis, 17 (74%) participants did not achieve 60 min MVPA on any monitoring day, and the remaining 6 participants engaged in ≥60 min MVPA on 3–5 days out of 7. Even in participants at the most functional ability level (GMFCS level I) we observed a highly sedentary lifestyle. The minimal amount of active time found in this pilot study highlights the dire need for intervention studies.

We believe the *ActiGraph* accelerometer is ready to use as a measure of habitual physical activity at home, at school, and in the community in children and adolescents with CP. The recent validation of cut-points for classification of activity levels in this population has provided a great opportunity to use accelerometers as an outcome measure in highly needed intervention studies that emphasize the integration of physical activity and participation into daily lifestyle. 

## 5. Conclusion

This study shows promise for using the *Actigraph *GT1M accelerometer as a feasible and meaningful measure in daily activity in adolescents with CP across GMFCS levels I–IV. 

## Figures and Tables

**Table 1:** Minutes of activity per hour and per day of monitoring time.

			GMFCS			
		Level I	Level II	Level III	Level IV	Total
		(*n* = 9)	(*n* = 5)	(*n* = 5)	(*n* = 4)	(*n* = 23)
LPA	min/day	121.5 (38.7)	95.7 (43.7)	66.7 (33.5)	32.1 (18.2)^∗^	88.4 (47.8)
	min/hr	10.2 (3.4)	8.2 (3.6)	5.6 (2.3)	1.6 (1.2)^∗†^	7.3 (4.2)
MPA	min/day	33.0 (15.6)	16.4 (12.5)	6.0 (5.7)^∗^	2.7 (3.7)^∗^	18.2 (17.1)
	min/hr	2.7 (1.2)	1.4 (1.0)	0.5 (0.4)^∗^	0.2 (0.3)^∗^	1.5 (1.4)
MVPA	min/day	56.0 (28.4)	26.6 (24.7)	9.3 (10.4)^∗^	5.8 (8.0)^∗^	30.7 (30.3)
	min/hr	4.5 (2.1)	2.2 (2.1)	1.5 (1.5)^∗^	0.5 (0.7)^∗^	2.6 (2.4)

Data are presented as mean (SD). ^∗^indicates significant difference from level I, ^†^indicates significant difference from level II, *P* < 0.05.

Minutes of activity per hour and per day of monitoring time.

LPA: light physical activity; MPA: moderate physical activity; MVPA: moderate-to-vigorous physical activity; CP: cerebral palsy; GMFCS: Gross Motor Function Classification System; min: minutes; hr: hour.

## Abbreviations

ANOVA: One-way analysis of variance
ANCOVA: Analyses of covariance
CP: Cerebral palsy
GMFCS: Gross Motor Function Classification System
GMFCS-E&R: Gross Motor Function Classification System, expanded and revised version
LPA: Light physical activity
MPA: Moderate physical activity
MVPA: Moderate-to-vigorous physical activity
PA: Physical activity
VPA: Vigorous physical activity.
